# Serum low-density lipoprotein and low-density lipoprotein expression level at diagnosis are favorable prognostic factors in patients with small-cell lung cancer (SCLC)

**DOI:** 10.1186/s12885-017-3239-z

**Published:** 2017-04-14

**Authors:** Ting Zhou, Jianhua Zhan, Wenfeng Fang, Yuanyuan Zhao, Yunpeng Yang, Xue Hou, Zhonghan Zhang, Xiaobo He, Yaxiong Zhang, Yan Huang, Li Zhang

**Affiliations:** 1 Department of Medical Oncology, Sun Yat-Sen University Cancer Center, 651 Dongfeng East Road, Guangzhou, 510060 People’s Republic of China; 2 State Key Laboratory of Oncology in South China, Guangzhou, 510060 People’s Republic of China; 3 Collaborative innovation Center for Cancer Medicine, Guangzhou, 510060 People’s Republic of China; 4 Department of Oncology, The Fifth Affiliated Hospital of Sun Yat-Sen University, Zhuhai, 519000 People’s Republic of China

**Keywords:** Small-cell lung cancer, Low-density lipoprotein, Low-density lipoprotein receptor, Prognosis

## Abstract

**Background:**

Patients with small-cell lung cancer (SCLC) patients demonstrate varied survival outcomes. Previous studies have reported that lipoproteins are associated with prognosis in various cancers; however, the role of low-density lipoprotein (LDL) and low-density lipoprotein- cholesterol (LDLR) in patients with SCLC has not been studied.

**Methods:**

In this study, the impact of LDL and LDLR on the prognosis of SCLC patients was evaluated. A total of 601 patients with SCLC were retrospectively evaluated, in which 198 patients had adequate tissues for immunohistochemistry, and serum LDL and LDLR expression levels at baseline were tested. X-tile tool, and univariate and multivariate Cox analysis were used to assess the association between LDL, LDLR and overall survival (OS).

**Results:**

Univariate analysis demonstrated that a lower LDL level was significantly associated with superior OS (*P* = 0.037). Similarly, LDLR also significantly predicted OS (*P* = 0.003). Multivariate Cox analyses confirmed that lower LDL and LDLR expression was independent prognostic factors associated with longer OS (*P* = 0.019 and *P* = 0.027, respectively).

**Conclusions:**

This study showed that both LDL and LDLR are prognostic indexes for survival in patients with SCLC. Patients with high LDL or LDLR expression level may benefit from treatment that modulates lipoprotein combined with platinum-based chemotherapy.

**Electronic supplementary material:**

The online version of this article (doi:10.1186/s12885-017-3239-z) contains supplementary material, which is available to authorized users.

## Background

Lung cancer remains the most common malignancy worldwide and accounts for the most cases of cancer related deaths in men and women [1]. Approximately 2.2 million new cases occur annually in the United States and 1.5 million people will die from this malignancy [2]. Up to 15% of newly diagnosed lung cancer in men and women are small-cell lung cancer (SCLC) [3–5]. SCLC is an aggressive subtype of lung cancer. About 60% of patients have extensive disease at diagnosis and many patients are at high risk for developing relapse disease [6]. Moreover, many patients with recurrent disease failed to respond effectively to chemotherapy due to developing resistance with treatment. The overall 5-year survival rate for SCLC patients with limited and extensive staging is 25 and 7.8%, respectively [7–9]. Therefore, patients with extensive SCLC have poor prognosis at initial diagnosis. Patients with SCLC patient have varied prognosis despite having similar staged disease. Therefore, identifying prognostic factors that are associated with clinical benefit may help guide treatment.

Cholesterol is a critical structural component of the cellular membrane in most cell types [10]. A number of studies have showed that cholesterol is associated with cell proliferation [11, 12], suggesting that abnormal cholesterol synthesis could play a role in the tumorigenesis of various tumor cells, including breast, colon, and nasopharyngeal [13–18]. The correlation between cholesterol and tumorigenesis in humans is currently an area of investigation; however, the mechanism by which abnormal cholesterol synthesis contributes to tumorigenesis remains unknown. Several studies have reported that cholesterol, particularly serum low-density lipoprotein (LDL), is abnormal in patients with cancer. Beyond the known functions of LDL as a key lipoprotein carrier of cholesterol, it is also a key factor in the signaling pathways of cancer cells [19]. Recently, LDL has been reported to promote cancer metastasis by regulating integrin transfer [20]. Otherwise, since tumor cells have more cholesterol requirements than normal cells, they may enhance their cholesterol content through receptor-mediated endocytosis of serum LDL by LDLR, which are able to recognize a series of ligands. Recent studies have demonstrated that low-density lipoprotein receptor (LDLR) play a role in cancer and is found overexpressed in various types of of human cancer cells [21, 22]. LDLR has also been reported to play an important role in tumor cancer growth and invasion by regulating NF-kB signaling [23].

Previous studies have indicated that LDL and LDLR are prognostic factors in pancreatic adenocarcinoma, which negatively correlated with clinical outcome [24]. However, the association of serum LDL and LDLR with clinical outcome in SCLC remained unknown. In this retrospective study, we explored the potential prognostic value of serum LDL and LDLR in SCLC patients. Moreover, we proposed that LDL and LDLR might be promising metabolic targets for anti-tumor therapy in SCLC.

## Methods

### Study population

This retrospective study involved data collection from SCLC patients between January 2004 to December 2011 at Sun Yat-Sen University Cancer Center (SYSUCC). All enrolled patients met the following criteria: (a) pathologically confirmed primary SCLC, (b) available clinical information, (c) normal liver function, and (d) detailed laboratory data, including cholesterol and LDL at diagnosis. In both groups, patients were recruited with lipid metabolism-related diseases, or currently treated for concomitant diseases that would influence serum lipids (i.e., diabetes, hyperlipidemia, or metabolic syndrome), patients with liver disease, or other types of cancer. A total 601 eligible patients were enrolled into the study. Among them, 198 cases have sufficient tumor specimens for immunohistochemistry (IHC). All patients were staged according to the Veterans Administration Lung Study Group (VALSG) staging system. Complete clinical information of all patients (i.e., demographics, performance status, treatments and laboratory tests) was recorded. Smokers were defined as patients who had more than 100 cigarettes. The study was approved by the Institutional Review Board of SYSUCC and written informed consent was obtained for each patient prior to sample collection.

### Treatment

Most patients received four cycles of platinum plus etoposide as chemotherapy, and some patients also were subsequently treated with prophylactic cranial irradiation (PCI). Several patients underwent thorax radiotherapy (TRT) in accordance with chemotherapy.

### LDLR immunohistochemistry and scoring

We performed IHC staining to evaluate the expression of LDLR in SCLC patients. Sections (thickness, 3–4 μm) were deparaffinized and rehydrated. For antigen retrieval, the slides were soaked in ethylene diamine tetraacetic acid (EDTA) and Aantigen Rretrieval Ssolution (3000 ml, pH 8.0), followed by heating in a pressure cooker for 12 min. Treated sections were then cooled to room temperature prior to immersing in distilled water for 2 min. To block the endogenous peroxidase activity and reduce non-specific assimilation, sections were treated with 3% H2O2 for 8 min, and further incubated in 5% bovine serum albumin for 30 min. Anti-LDLR (mouse LDLR antibody; R&D Systems; American) (1:400 dilution) was then added and incubated at 4 °C for 24 h. After washing with phosphate-buffered saline (PBS) for three cycles of 2 min, slides were incubated with secondary antibody (PV-9003 goat kit; ZSGB-Bio, Beijing, China) at 37 °C for 30 min. Afterwards, slides were washed with PBS thrice again. 3, 3′- diamino benzidine was applied for dyeing and hematoxylin was used to counterstain the sections. All sections were independently reviewed by two pathologists. Semi-quantitative scoring was used to evaluate immune reaction [25]. An IHC score, called HSCORE, was then applied to each sample based on the intensity of staining and the percentage of positive tumor cells. The HSCORE was calculated as following: HSCORE = Ʃ (I × PC). “I “means the intensity of staining and “PC” represents the percentage of positive tumor cells.

### Follow-up

All the patients were carefully followed. Patients were evaluated every 2 months after completion of anti-tumor therapy. Routine follow-up examination was performed by computed tomography (CT) scan or/and Magnetic Resonance Imaging (MRI), including chest radiograph, abdominal ultrasonography and brain when clinically indicated. Anti-tumor response was assessed by radiologists according to the Response Evaluation Criteria in Solid Tumors (RECIST, version 1.1). Overall survival (OS) was defined as the months from the diagnosis to the death for any cause or last follow-up. Progression-free survival (PFS) was defined as the months from the diagnosis to the earliest occurrence of disease progression or death for any reasons. Patients who were alive at the time of last follow-up or lost to follow-up were censored. The last follow-up date was determine at May 31, 2015.

### Statistical analysis

The primary outcome of the study was overall survival. Pearson correlation, Chi-square test, and Fisher exact test were used to compare continuous and categorical variables. The optimal cutoff values of LDL and LDLR level were determined using X-tile. Kaplan-Meier method was performed to estimate the relationship between overall survival (OS) and potential prognostic factors. Univariate analysis was performed to assess differences in survival by log-rank test. Cox proportional hazards model was used to estimate the predictive power. Potential prognostic factors included in the test model were age, sex, performance status (PS), cancer stage, LDL and LDLR. A *P* value of ≤0.05 was considered statistically significant. All of the statistical tests were two-tailed. Data analyses were carried out using the SPSS statistical software package (version 21.0, IBM, Armonk, NY).

## Results

### Patient characteristics

The patient baseline characteristics are presented in Table 1. A total of 601 patients with SCLC were enrolled in the study, with a median age of 60 years (range, 19–82 years). The majority of the patients were males (*n* = 529, 88%) and smokers (*n* = 505, 84.0%), and had a PS of 0–1 (*n* = 550, 91.7%). Among them, 254 (42.3%) patients had distant metastasis at the time of diagnosis and 347 (57.7%) patients were at limited stage. Most of the patients had prior treatment of two to four cycles of etoposide-based chemotherapy, while 22.1% (*n* = 133) patients received PCI and 37.8% (*n* = 227) had TRT. At last follow-up date, 433 (72%) patients had died. The median follow-up time was 31.75 months (range, 3.32 months to 117.41 months).

**Table 1:** Basic characteristic of all patients for 601 patients with SCLC

Variables	All cohort	
	No.	Percent
Age (years)		
Median	60.0	
Range	19.0–82.0	
Gender		
Female	529	88.0
Male	72	12.0
Cancer stage		
Limited stage	347	57.7
Extensive stage	254	42.3
Smoking status		
Never	505	84.0
Current or Ever	95	16.0
PS		
0	311	51.7
1	239	39.8
2	50	8.3
PCI		
Yes	133	22.1
No	468	77.9
Chemotherapy		
Etoposide-based	498	82.9
Others	102	17.0
TRT		
Yes	227	37.8
No	374	62.2
LDL		
Low	66	11.0
Intermediate	282	46.9
High	253	42.1

*Abbreviations SCLC* small-cell lung cancer, *PS* performance status, *PCI* prophylactic cranial irradiation, *TRT* thorax radiotherapy, *LDL* low-density lipoprotein

### Correlation of LDL and LDLR with clinical features

Using X-tile [26], we determined that the optimal cutoff for serum LDL in assessing OS is 2.14 and 3.36. Patients were divided into three groups based on the cutoff value of LDL: (1) low-LDL group (LDL level ≤ 2.14 mmol/L, *n* = 66, 11.0%) (2) intermediate-LDL group (3.36 mmol/L < LDL level ≥ 2.14 mmol/L, *n* = 282, 46.9%) and (3) High-LDL group (LDL level > 3.36 mmol/L, *n* = 253, 42.1%) (Additional file 1: Figure S1).

The clinicopathological characteristics of SCLC patients based on LDL levels are presented in Table 2. More patients had low levels of serum LDL in the etoposide-based chemotherapy group compared with those in the non-etoposide-based chemotherapy group (*P* = 0.011, Table 2). However, the level of LDL was not significant associated with age (*P* = 0.648), gender (*P* = 0.918), PS (*P* = 0.119), smoking status (*P* = 0.411), and disease stage (*P* = 0.189) (Table 2).

**Table 2:** Association of the LDL level with clinical characteristics

Variables	Low LDL		Intermediate LDL		High LDL		*P-value*
	No.	%	No.	%	No.	%	
Patients							
Age (years)							*0.648*
19–60	36	11.3	144	45.1	139	43.6	
61–82	30	10.6	138	48.9	114	40.4	
Gender							*0.918*
Female	7	9.7	35	48.6	30	41.7	
male	59	11.2	247	46.7	223	42.2	
Disease stage							*0.189*
Limited stage	45	13.0	160	46.1	142	40.9	
Extensive stage	21	8.3	122	48.0	111	43.7	
Smoking status							*0.411*
Never	9	9.5	52	54.7	34	35.8	
Current or Ever	51	11.3	229	45.3	219	43.4	
Chemotherapy							*0.011*
Etoposide-based	60	12.0	228	45.8	210	42.2	
Other	5	4.9	54	52.9	43	42.2	
PS							*0.119*
0	28	9.0	152	48.9	131	42.1	
1	35	14.6	105	43.9	99	41.4	
2	2	10.8	25	50.0	23	46	
PCI							*0.092*
Yes	10	7.5	57	42.9	66	49.6	
No	456	12.0	225	48.1	187	40.0	
TRT							*0.546*
Yes	24	10.6	101	44.5	102	44.9	
No	42	11.2	181	48.4	151	40.4	

*Abbreviations LDL* low-density lipoprotein, *PS* performance status, *PCI* prophylactic cranial irradiation, *TRT* thorax radiotherapy

**Fig. 1:**
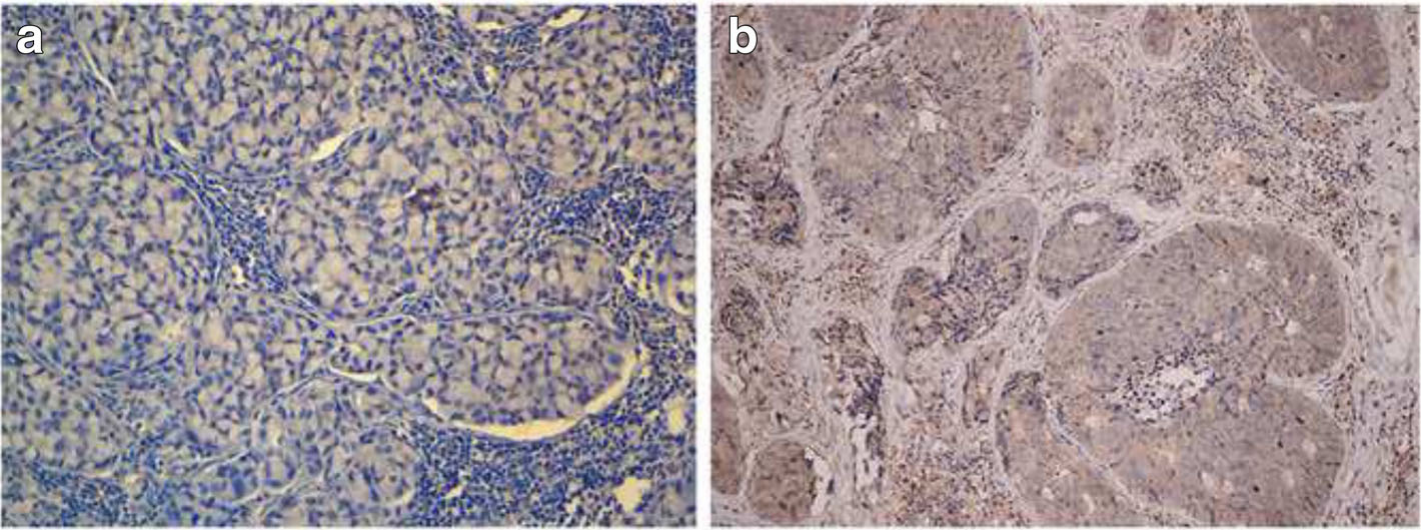
Representative images of immunostaining of LDLR expression based on different levels of expression (Original magnification 200×). **a** Low LDLR expression, **b** High LDLR expression. LDLR, low-density lipoprotein

**Fig. 2:**
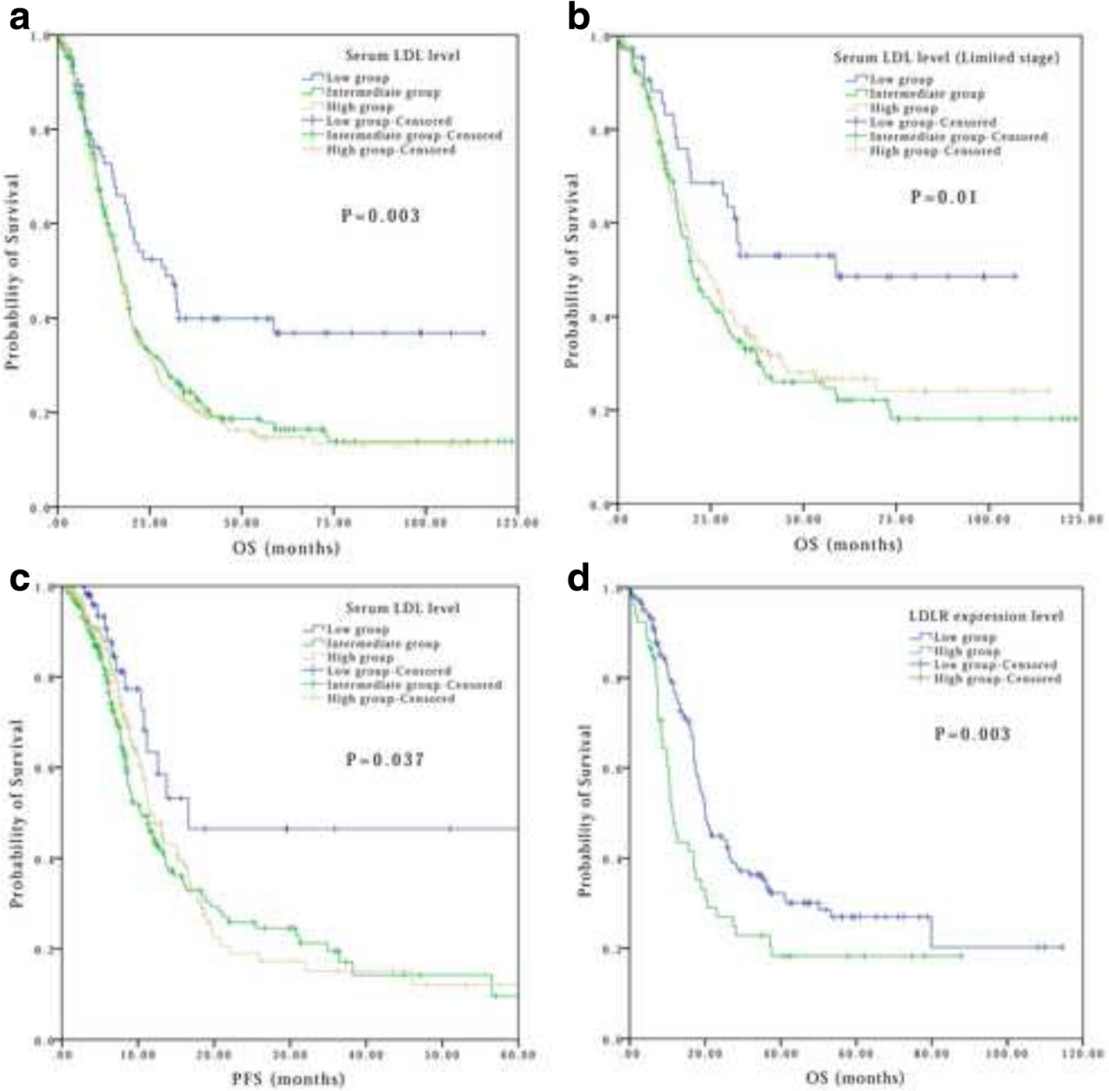
Kaplein Meyer survival curves for patients with SCLC based on LDL levels. **a** Comparison of OS in overall patients based on LDL levels, **b** Comparison of OS in patients with limited stage based on LDL levels, **c** Comparison of PFS in patients based on LDL levels, **d** Comparison of OS in patients based on LDLR expression level. LDL: low-density lipoprotein; LDLR: low-density lipoprotein

Based on the results by X-tile, the 198 patients who had sufficient tumor samples for IHC were grouped as followed: (1) low-LDLR group (HSCORE ≤ 60, *n* = 145, 73.2%, Fig. 1a), and (2) high-LDLR group (HSCORE >60, *n* = 53, 26.8%, Fig. 1b) (Additional file 1: Figure S1).

Among the 198 patients, 175 ones were males, ones were smokers, and ones had a PS of 0–1. The relationship between LDLR and clinical features have been analyzed. There were no obvious correlation of LDLR to gender (*P* = 0.565), PS (*P* = 0.118), and smoking status (*P* = 0.069).

### Univariate Cox regression analysis of survival

The median OS for the 601 eligible patients was 15.43 months (range, 0.03–123.43 months). The median PFS for the entire cohort was 5.32 months (range, 0.03–71.79 months). A total of 66, 282, and 253 patients were categorized as low-LDL, intermediate-LDL, and high-LDL groups. Compared with the low-LDL group, patients with intermediate-LDL or high-LDL had lower survival outcome (low-LDL vs. intermediate-LDL vs. high-LDL, 29.27 vs. 16.70 vs. 17.23 months, respectively; *P* = 0.003) (Fig. 2a). When stratified by cancer stage, we found that LDL also showed a prognostic power in limited stage (*P* = 0.01, Fig. 2b). Moreover, baseline serum LDL value also had distinct significance in predicting PFS (*P* = 0.037, Fig. 2c).

To provide a significant control and a point of reference for LDLR expression, we also study the immune-staining of healthy lung tissue. The image shows that the expression of LDLR in healthy lung tissue is very low (Additional file 2: Figure S2). Moreover, patients with a lower expression of LDLR demonstrated significantly better OS (19.94 vs. 11.27 months, respectively; *P* = 0.003, Fig. 2d).

**Fig. 3:**
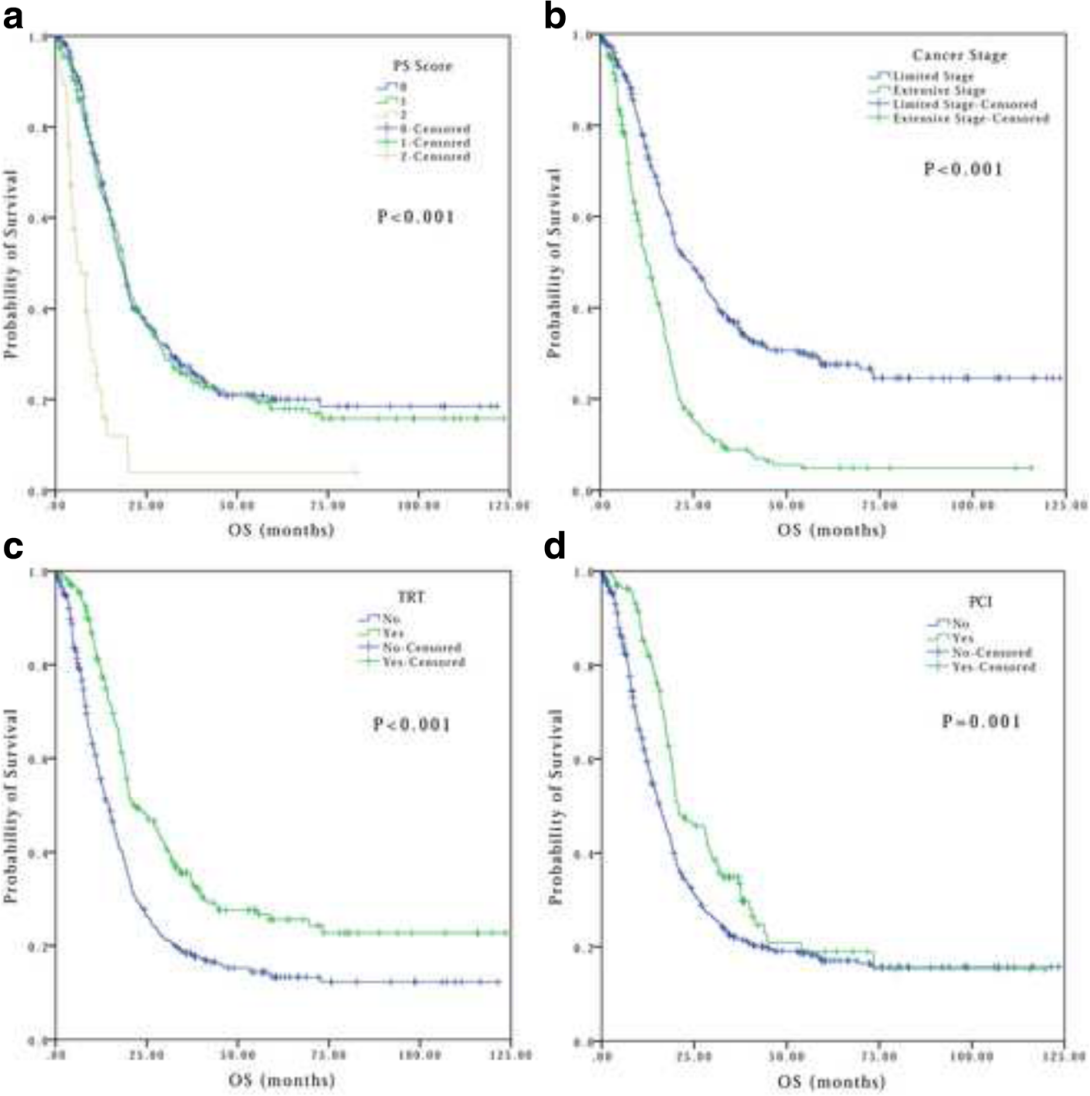
Overall survival curves of patients with SCLC **a** Good vs bad PS, **b** Limited stage vs extensive stage, **c** Received TRT vs none, **d** Received PCI vs none. PS: performance status; TRT: thorax radiotherapy; PCI: prophylactic cranial irradiation

Other than LDL and LDLR, PS score (*P* < 0.001), smoking status (*P* < 0.001), and disease stage (*P* < 0.001) were also significantly associated with OS. Patients who received TRT (*P* < 0.001) or PCT (*P* = 0.001) also were associated with better OS (Fig. 3). However, there were no distinct associations between OS and gender (*P* = 0.438) and age (*P* = 0.424) (Table 1). In addition, patients with a lower PS score and in limited stage demonstrated significantly better PFS (*P* = 0.002 and *P* < 0.001, respectively).

### Multivariate Cox regression analysis of potential prognostic factors

Multivariate analyses, was performed to test for correlation among the different prognostic variables. We observed that higher LDL level was a significantly independent prognostic factor for poorer OS (*P* = 0.019, Table 3). Patients in intermediate LDL group were estimated to have 1.42-fold higher risk of death compared with those in the low LDL group (HR, 1.42; 95% CI: 1.08–2.03; *P* = 0.015). Patients with a LDL level of >3.36 had 1.64-fold higher risk of death than those in the low LDL group. Similarly, the multivariate analyses demonstrated that LDLR expression independently predicted OS in patients with SCLC (*P =* 0.027, Table 3). Compared with patients with a HSCORE ≤ 60, those with a HSCORE >60 had a 1.538 higher risk of death. Moreover, PS score (*P* < 0.001), cancer stage (*P* < 0.001), PCI (*P* = 0.011), and TRT (*P* = 0.007) were also independent predictors of survival outcome in patients with SCLC (Table 3).

**Table 3:** Univariable and multivariable analyses of potential prognostic factors in SCLC patients

Predictors	Univariable analysis			Multivariable analysis		
	HR	95% CI	*P-value*	HR	95% CI	*P-value*
Gender	-		0.438	-	-	0.118
Female	1 (Referent)	-		1 (Referent)	-	-
Male	0.89	.66–1.20		0.74	0.51–1.08	-
Age (years)	-		0.424			0.597
≤ 60	1 (Referent)	-		1 (Referent)	-	-
> 60	1.08	0.89–1.30		1.05	0.87–1.27	-
Cancer-stage	-		<0.001			<0.001
limited stage	1 (Referent)	-		1 (Referent)	-	-
Extensive stage	2.39	1.97–2.90		2.30	1.88–2.81	-
Smoking status	-		0.122			0.126
Never	1 (Referent)	-		1 (Referent)	-	-
Current or Ever	1.22	0.95–1.57		1.28	0.93–1.76	-
PS	-		<0.001			<0.001
0	1 (Referent)	-		1 (Referent)	-	-
1	1.04	0.85–1.27		0.98	0.80–1.19	-
2	3.79	2.64–5.40		3.62	2.52–5.20	-
Chemotherapy	-		0.386			0.96
Etoposide-based	1 (Referent)	-		1 (Referent)	-	-
Others	1.50	0.21–10.81		1.05	0.15–7.63	-
PCI	-		<0.001			0.011
Yes	1 (Referent)	-		1 (Referent)	-	-
No	1.46	1.15–1.84		1.38	1.08–1.77	-
TRT	-		<0.001			0.007
Yes	1 (Referent)	-		1 (Referent)	-	-
No	1.73	1.42–2.11		1.34	1.08–1.66	-
LDL	-		0.003			0.019
Low	1 (Referent)	-		1 (Referent)	-	-
Intermediate	1.73	1.22–2.45		1.42	1.00–2.03	-
High	1.81	1.28–2.58		1.64	1.15–2.35	-
LDLR	-		0.003			0.027
Low	1 (Referent)	-		1 (Referent)		
High	1.61	1.11–2.34		1.54	1.05–2.26	

Abbreviations *SCLC* small-cell lung cancer, *PS* performance status, *LDL* low-density lipoprotein, *PCI* prophylactic cranial irradiation, *TRT* thorax radiotherapy, *LDLR* low-density lipoprotein receptor

## Discussion

Cholesterol plays a critical role in maintaining the structural integrity of the plasma cell membrane [27, 28]. In addition, cholesterol also accumulates in specific domains of the membrane and associates with proteins that are involved in various cellular signaling pathways [29]. A study by Guillaumond et al. found that cholesterol uptake is significantly increased in pancreatic adenocarcinoma [24]. Several studies indicated that cholesterol modulates the development and progression of various cancers [30, 31]. Moreover, a recent study identified that cholesterol could be a prognostic index for patients with metastatic nasopharyngeal carcinoma [17].

LDL is a component of cholesterol and is involved in cholesterol transportation. A recent study has indicated that LDL level is associated with increased risk of developing hepatocellular carcinoma [32]. Rodrigues et al. demonstrated that LDL level was an adverse predictor of disease-free survival in breast cancer patients [33]. In CRC patients, LDL was also identified as an independent prognostic factor [34]. Nevertheless, the mechanism by which LDL levels are associated with cancer development remains unclear. LDLR, a receptor for LDL, can activate signaling pathways involved in inflammation, cellular transformation, and cell growth. Previous studies demonstrated that LDLR has a pro-tumorigenic effect [35]. Studies have also demonstrated that the expression of LDLR in tumor cells is higher than in normal cells, and has been reported to promote cancer progression by increasing proliferation and migration of tumor cells [24, 36, 37].

Although studies have suggested a significant relationship between LDL, LDLR and cancer, levels of LDL and LDLR expression are varied across patients of different tumor types. Thus, in this study, we assessed the prognostic impact of LDL and LDLR expression levels in SCLC patients. To our knowledge, this is the first large-scale cohort study to explore the prognostic value of LDL and LDLR levels in SCLC patients. Based on cutoff value of LDL levels at diagnosis, we observed that 89% (*n* = 535) of SCLC patients had elevated serum LDL. We next evaluated the effects of LDL levels on OS. Univariate analysis demonstrated that high levels of LDL were associated with poorer survival in SCLC patients. Consistently, multivariate analysis demonstrated that LDL was also an independent prognostic factor in SCLC. Our study also suggested that lower LDLR was significantly associated with longer OS, compared with higher LDLR. Additionally, we showed that LDLR is also an independent predictor for OS. The stratification of patients according to disease stage showed that LDL level is a predictor of limited stage and not extensive stage. While the reason for this observation remains unclear, we speculate that an abnormal metabolic microenvironment in patients with extensive disease may influence LDL levels. Low LDL level was a predictor of longer PFS, which was consistent with the findings by previous published studies.

Based on the findings of this study, we speculate that higher serum LDL and LDLR expression level could be attributed to active tumor cells secreting high levels of cholesterol. Therefore, our findings support the notion that lipoprotein treatment may be a promising anti-tumor agent in patients with high LDL or LDLR expression level. Moreover, combination therapies of LDL-lowering agent with platinum-based chemotherapy may improve clinical outcome of patients. Nonetheless, there remains a need for identifying effective agents to improve the clinical outcomes of patients with SCLC, such as evaluating whether LDLR is a potential therapeutic target.

Our study does have several limitations. First, it is a retrospective study with clinical data primarily derived from a single institution. Future studies will involve patients from multiple centers to validate our findings. Second, it remains unclear the mechanism by which increased LDL level occurs in patients with SCLC. Additional studies will be needed to elucidate this.

## Conclusions

In summary, our results suggest that the serum LDL and LDLR expression level at diagnosis could serve as a significant prognostic factor in patients with SCLC. Serum LDL and LDLR expression in tumor cells at diagnosis could help identify patients susceptible to disease progression. Furthermore, the development of LDL-lowering agents combined with platinum-based chemotherapy may be a new and promising therapeutic strategy for SCLC patients. Therefore, baseline LDL and LDLR expression level could be routinely applied to guide treatment decisions in patients with SCLC.

## Additional files

Additional file 1: Figure S1. Bar plots of baseline serum LDL and LDLR expression levels of the SCLC patients base on the cutoff values. (TIFF 3195 kb)

Additional file 2: Figure S2. Representative image of immunostaining of LDLR expression in healthy lung tissue (Original magnification 200×). (TIFF 6774 kb)

## Abbreviations

CT: Computed tomography
EDTA: Ethylene diamine tetraacetic acid
IHC: Immunohistochemistry
LDL: Low-density lipoprotein
LDLR: Low-density lipoprotein- cholesterol
MRI: Magnetic Resonance Imaging
OS: Overall survival
PBS: Phosphate-buffered saline
PCI: Prophylactic cranial irradiation
PFS: Progression-free survival
PS: Performance status
RECIST: Response Evaluation Criteria in Solid Tumors
SCLC: Small-cell lung cancer
SYSUCC: Sun Yat-Sen University Cancer Center
TRT: Thorax radiotherapy
VALSG: the Veterans Administration Lung Study Group
