# Supplementary figures and images for: Serum low-density lipoprotein and low-density lipoprotein expression level at diagnosis are favorable prognostic factors in patients with small-cell lung cancer (SCLC)
Source: BMC Cancer. 2017 Apr 14;17:269. doi: 10.1186/s12885-017-3239-z (PMC5391547; doi:10.1186/s12885-017-3239-z)

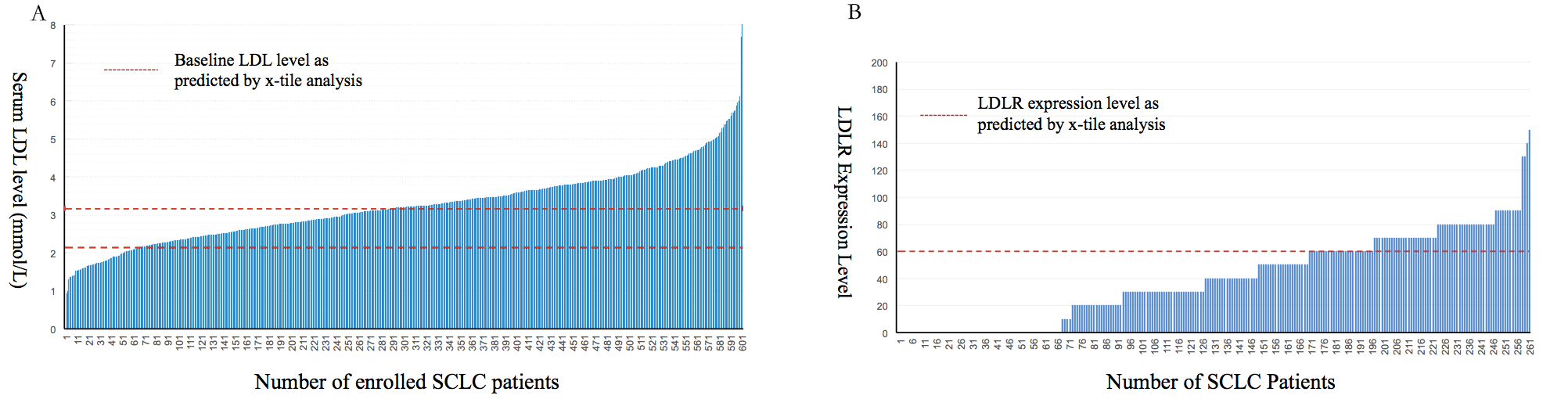

Supplement: Supplementary file 1 — Bar plots of baseline serum LDL and LDLR expression levels of the SCLC patients base on the cutoff values. (TIFF 3195 kb) [file 12885_2017_3239_MOESM1_ESM.tif]

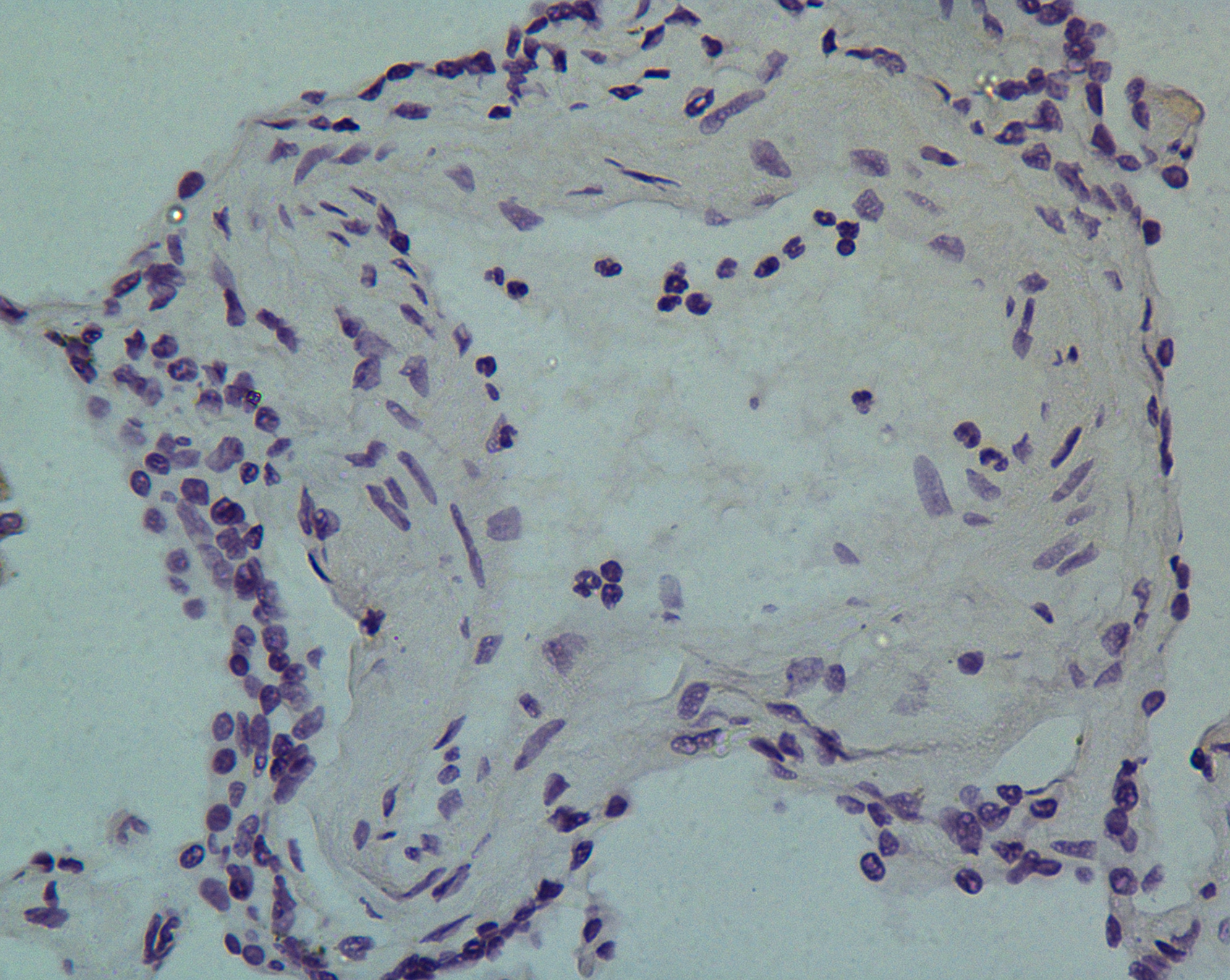

Supplement: Supplementary file 2 — Representative image of immunostaining of LDLR expression in healthy lung tissue (Original magnification 200×). (TIFF 6774 kb) [file 12885_2017_3239_MOESM2_ESM.tif]
